# Coupled changes in western South Atlantic carbon sequestration and particle reactive element cycling during millennial-scale Holocene climate variability

**DOI:** 10.1038/s41598-021-03821-8

**Published:** 2021-12-21

**Authors:** Bruna B. Dias, Alexander M. Piotrowski, Cátia F. Barbosa, Igor M. Venancio, Cristiano M. Chiessi, Ana Luiza S. Albuquerque

**Affiliations:** 1 Departamento de Geoquímica, Universidade Federal Fluminense, Niterói, 24020-141 Brazil; 2 School of Arts, Sciences and Humanities, University of São Paulo, São Paulo, 03828-000 Brazil; 3 Godwin Laboratory for Palaeoclimate Research, Department of Earth Sciences, University of Cambridge, Cambridge, CB2 3EQ UK; 4 Center for Weather Forecasting and Climate Studies (CPTEC), National Institute for Space Research (INPE), Cachoeira Paulista, 12630-000 Brazil; 5 MARUM - Center for Marine Environmental Sciences, University of Bremen, Leobener Strasse, D-28359 Bremen, Germany

**Keywords:** Carbon cycle, Element cycles, Palaeoceanography, Palaeoclimate

## Abstract

Continental shelves have the potential to remove atmospheric carbon dioxide via the biological pump, burying it in seafloor sediments. The efficiency of marine carbon sequestration changes rapidly due to variations in biological productivity, organic carbon oxidation, and burial rate. Here we present a high temporal resolution record of marine carbon sequestration changes from a western South Atlantic shelf site sensitive to Brazil Current-driven upwelling. The comparison of biological records to rare earth element (REE) patterns from authigenic oxides shows a strong relationship between higher biological productivity and stronger particle reactive element cycling (i.e. REE cycling) during rapid climate change events. This is the first evidence that authigenic oxides archive past changes in upper ocean REE cycling by the exported organic carbon. In addition, our data suggest that Brazil Current-driven upwelling varies on millennial-scales and in time with continental precipitation anomalies as registered in Brazilian speleothems during the Holocene. This indicates an ocean–atmosphere control on the biological pump, most probably related to South American monsoon system variability.

## Introduction

The production and burial of marine organic carbon play a key role in oceanic biogeochemical processes at shallow and intermediate depths. Primary producers in the surface ocean (i.e. phytoplankton) uptake carbon dioxide that ultimately comes from the atmosphere to produce organic carbon and support the column water food web. The proportion of soft tissue that escapes degradation in the water column and is buried has been termed “marine carbon sequestration”^1^. The effectiveness of carbon sequestration in the shallow benthic environment is limited by the biological production, oxidation, and burial rate of organic material^2^. Despite the achievements in the understanding of the role of recent climate change in carbon sequestration^3^, it is still poorly known to what extent natural centennial to millennial-scale climate variability of the Holocene (11.7 ka ago to present) impacted carbon sequestration.

Rapid climate changes during the Holocene have been recorded globally on centennial to millennial-scale. They are especially prevalent and well-studied in the North Atlantic through records of freshwater influx and sea-surface temperature (SST)^4,5^, as well as its ecological impacts over the deep-sea biodiversity^6^. The cause of these changes however remain unknown, but changes in solar activity, oceanic and atmospheric heat transport have been proposed^7^. The interhemispheric pattern of rapid centennial to millennial-scale variability during the Holocene seems to exhibit a “seesaw” behavior, as expressed in the North and South Atlantic. During rapid cold conditions in the North Atlantic, western South Atlantic SST exhibit anomalously high temperatures because of the decreased northward oceanic heat transport^8^. The rapid changes observed in Southern Hemisphere climatic records suggests that the South American Monsoon System (SAMS) and the western South Atlantic surface ocean circulation varied at same timing^8–11^. Both SAMS and the South Atlantic surface circulation are sensitively linked to North Atlantic variability by the Intertropical Convergence Zone (ITCZ), which shows a seasonal meridional migration and a latitudinal contraction following the orbital-scale insolation-driven changes^12^.

During the austral summer, when ITCZ is southward displaced, intensified wind stress causes the Brazil Current (BC) to strengthen^13^ and drives physical oceanographic changes along the Brazilian margin. A strong BC fosters the upwelling of South Atlantic Central Waters (SACW) in specific zones of the Brazilian margin, bringing nutrients to the surface and stimulating biological primary productivity. This strengthening has been observed in instrumental datasets^14^, recent records^15,16^, and Holocene reconstructions^17^. However so far there has been no high temporal resolution record focusing in compare centennial to millennial-scale Holocene changes in the biological pump to the rapid Northern Hemisphere climate events.

Marine carbon sequestration is closely related to the flux of particulate organic carbon through the water column. Particle flux also controls the distribution of particle reactive trace elements, in particular the fractionation of the rare earth elements (REE). Recent works have suggested that particulate organic carbon can cycle particle reactive trace elements such as REE^18–22^, confirming the importance of continental shelves as potential REE sink^23^. Scavenging by organics also opens raises the possibility that carbon and REE cycles are closely related, opening the possibility of using REE as proxies for carbon sequestration in the ocean. Here we investigate Holocene climate forced export of particulate organic carbon using a benthic foraminiferal productivity record and compare this to REE patterns measured on sedimentary foraminifera with authigenic oxides (i.e. foraminiferal coatings) from a sediment core collected at the continental shelf of the western South Atlantic. The biological and chemical association were applied to assess the potential of authigenic oxides to record the exported organic carbon control over REE cycling during rapid climate change events.

### Study area

**Figure 1:**
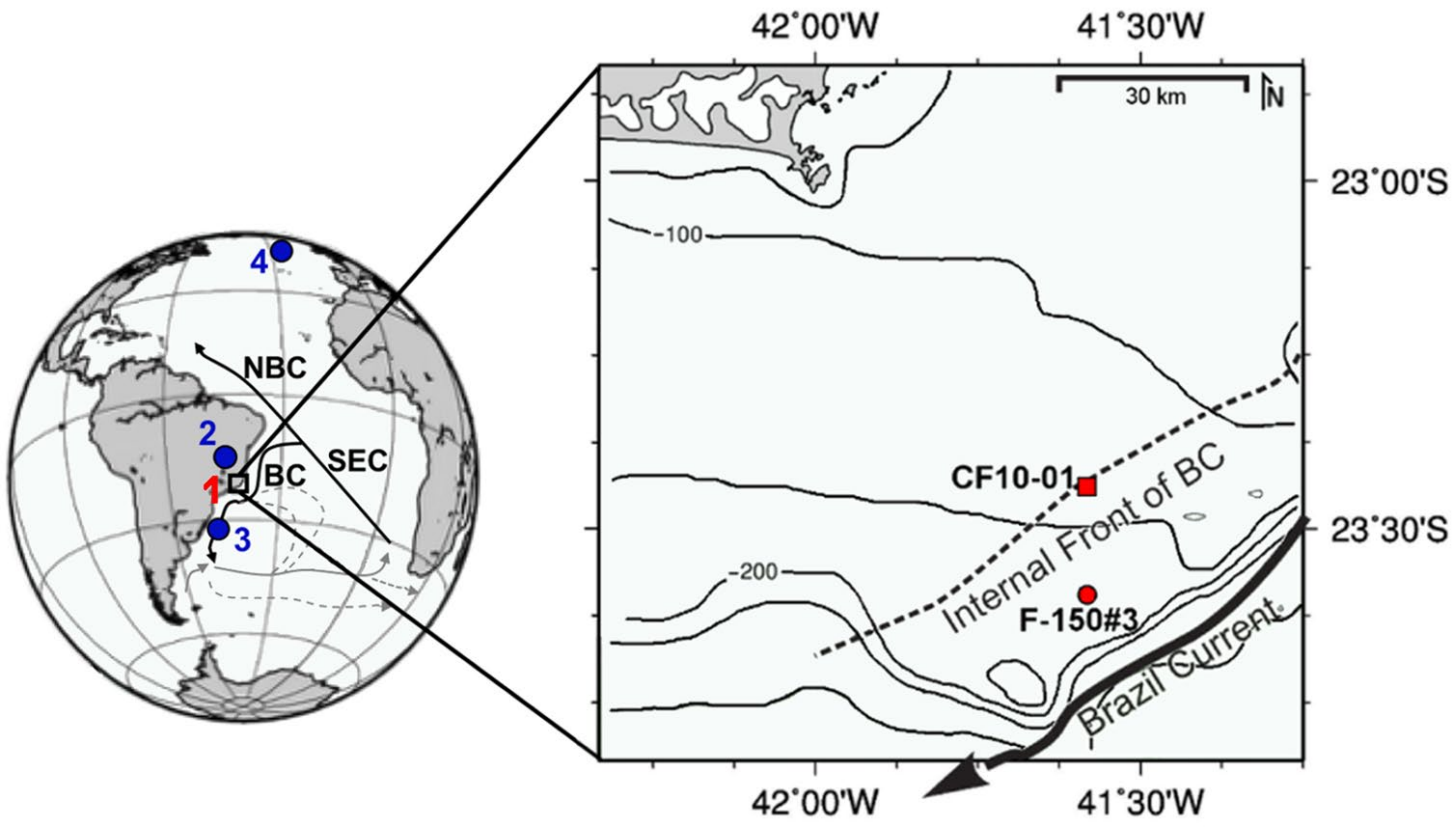
Location of Cabo Frio Upwelling System (squared inset) and its oceanographic characteristics, including the inshore (dashed line) and offshore (unbroken bold arrow) positions of BC. The following records are shown: (1) marine sediment core CF10-01 (red square) and sediment trap F-150#3 (red circle) (this study); (2) speleothems from Lapa Grande and Diva cave speleothems^10,11^; (3) marine sediment core GeoB6211-1/2^8^; (4) marine sediment core LO09-14^5^. BC: Brazil Current, NBC: North Brazil Current, SEC: South Equatorial Current. Bathymetric lines corresponding to 50–300 m depth. The map was generated with GMT version 4 (https://www.generic-mapping-tools.org/) and edited in GIMP version 2 (https://www.gimp.org/).

The Cabo Frio Upwelling System (CFUS) is located off the southeastern Brazil and is influenced by the oligotrophic western boundary BC (Fig. 1). The BC originates from the bifurcation of the South Equatorial Current, resulting in the northward-flowing North Brazil Current and the southward-flowing BC. The BC carries warm and nutrient-poor waters at shallow depths (Tropical Water), as well as cold and nutrient-rich waters of the SACW at thermocline depths^24^.

Our study location is sensitive to BC-driven upwelling at the shelf break and uppermost slope, but not the Ekman transport observed in the inner continental shelf of CFUS. BC-driven upwelling is characterized by SACW pumping into the photic zone by the wind stress curl and the BC offshore displacement away from its inshore position (Internal Front of BC) during the austral summer (strong BC)^25^ (Fig. 1). The SACW intrusion boosts primary productivity^26,27^ and promotes the accumulation of autochthonous marine organic carbon^28,29^, making the CFUS a hotspot for carbon sequestration and storage in the oligotrophic western South Atlantic. Near the CFUS, the Paraíba do Sul River potentially discharge terrestrial sediments from the Ribeira belt^30^ to the continental shelf, which are prone to be carried southward by the BC^31,32^.

Increases in the BC strength are also coincident with increased SST in the western tropical South Atlantic^13^. The BC strengthening and its displacement far away from the continental shelf break is in agreement with the SST anomalies observed during the Holocene^8,17^. Besides, the SST anomalies in the southeastern Brazilian margin have been related to the intensification of the South Atlantic Convergence Zone over Southeastern South America, a component of the SAMS^33^.

## Results

### Elemental composition of foraminifera and biological productivity

**Figure 2:**
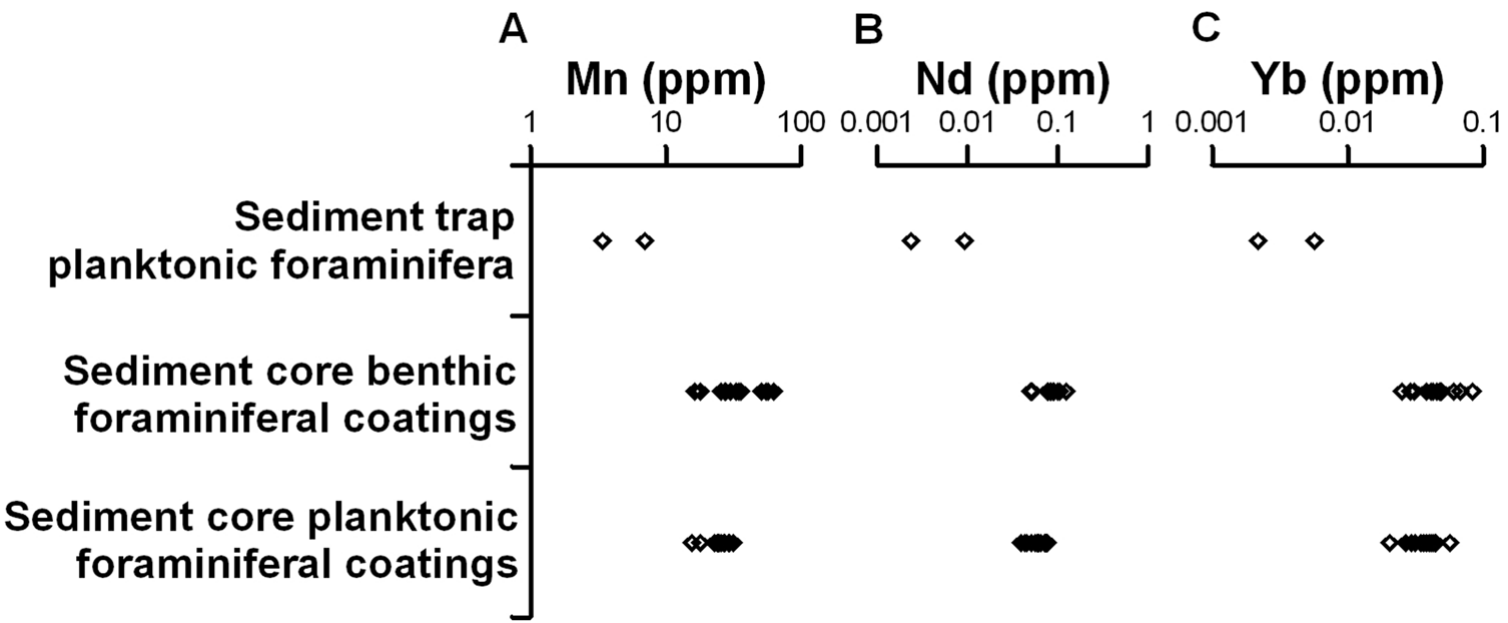
Concentration of trace and REE in planktonic foraminifera from sediment trap F-150#3 and foraminiferal authigenic oxide coatings from core CF10-01. **(A)** Mn concentration (ppm), **(B)** Nd concentration (ppm), and **(C)** Yb concentration (ppm) in planktonic foraminifera from sediment trap, planktonic foraminiferal coatings from sediment core, and benthic foraminiferal coatings from sediment core. Note the logarithmic scale.

Low Mn and REE concentrations are observed in sediment trap planktonic foraminifera in comparison to planktonic and benthic sedimentary foraminifera with authigenic oxide coatings. The comparison between sedimentary planktonic and benthic foraminifera with authigenic oxide coatings showed slight differences in the Mn (*t* = 2.87; *p* < 0.01) and Nd (*t* = 3.69*; p* < 0.01) concentrations. Benthic foraminifera with coatings have slightly higher average trace elements concentrations (Mn = 35.56 ± 14.21 ppm; Nd = 0.08 ± 0.02 ppm) than planktonic foraminifera with coatings (Mn = 24.52 ± 4.48 ppm; Nd = 0.06 ± 0.01 ppm) (Fig. 2). These results imply that the Mn and REE are associated with the authigenic oxides precipitated onto the foraminifera shells as a *post-mortem* process.

**Figure 3:**
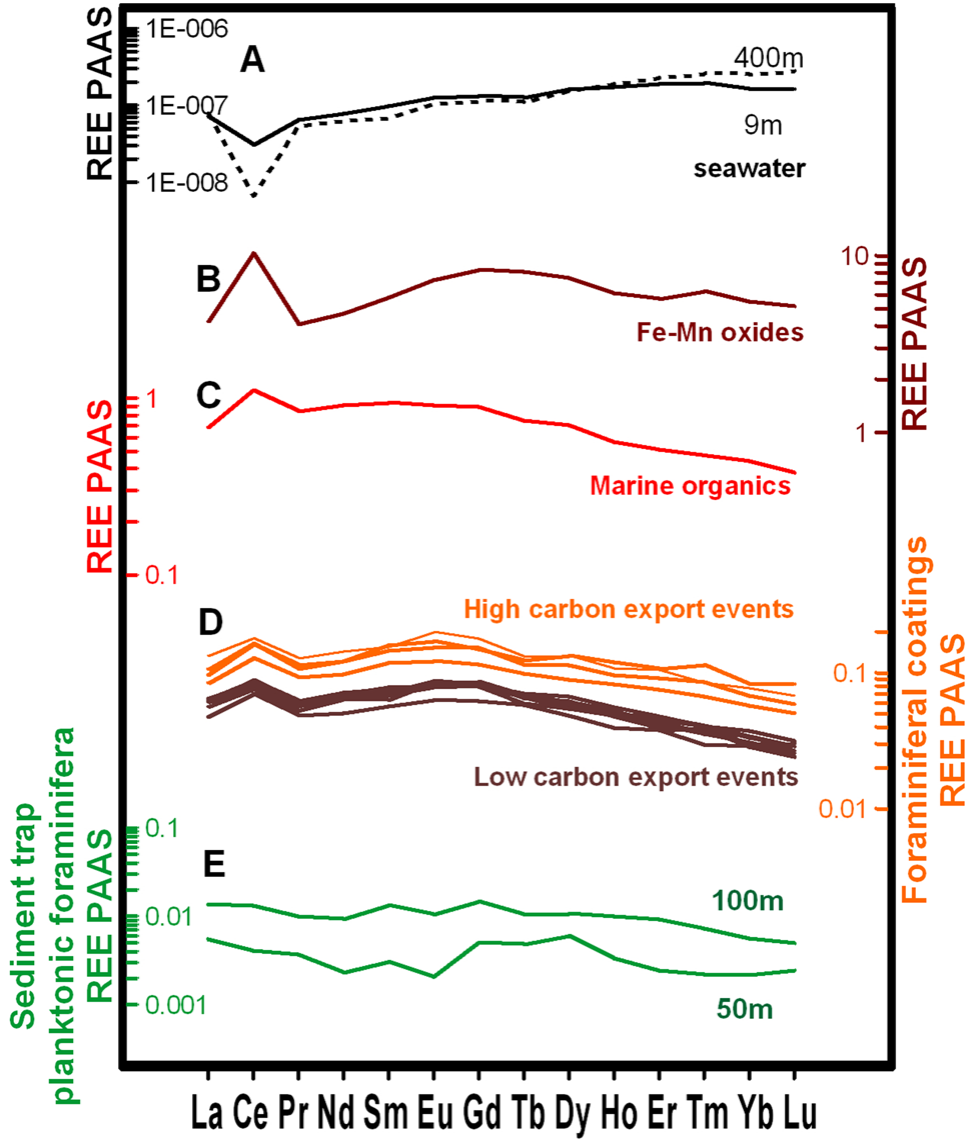
Post-Archaean Australian Shale (PAAS) normalized REE patterns for: **(A)** Brazil Basin seawater from 9 and 400 m water depth at 11^o^ S^42^ (black continuous and dashed lines); **(B)** average of Fe–Mn oxides^19^ (dark red); **(C)** average of marine organic carbon from the Atlantic Ocean^19^ (light red); **(D)** foraminiferal authigenic oxide coatings from core CF10-01 (orange and brown lines; this study); and **(E)** planktonic foraminifera from sediment trap F-150#3 (green lines; this study).

**Figure 4:**
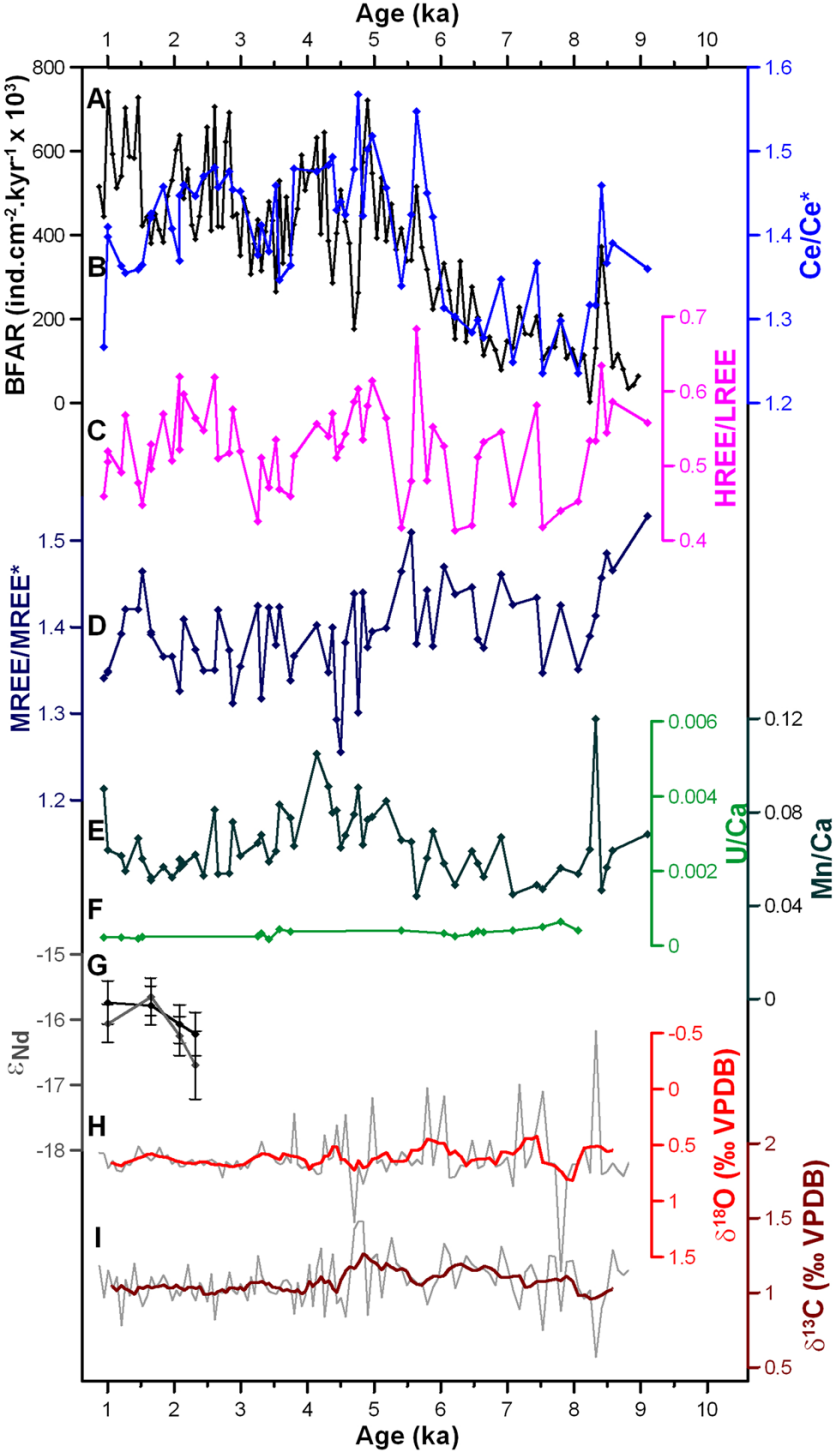
Records of organic carbon export, trace element concentrations, and isotopic composition of foraminifera from core CF10-01. **(A)** BFAR record (black; this study), **(B)** Ce anomaly (Ce/Ce*) of benthic foraminiferal coatings (light blue; this study), **(C)** HREE/LREE of benthic foraminiferal coatings (purple; this study), **(D)** Middle-REE enrichment (MREE/MREE*) of benthic foraminiferal coatings (dark blue; this study), **(E)** Mn/Ca of benthic foraminiferal coatings (dark green; this study), **(F)** U/Ca of benthic foraminiferal coatings (light green; this study), **(G)** *ε*_Nd_ of benthic (black) and planktonic (gray) foraminiferal coatings (this study), **(H)** δ^18^O of epibenthic foraminifera *Cibicides kullenbergi* (light red; this study), and **(I)** δ^13^C of epibenthic foraminifera *C. kullenbergi* (dark red; this study).

The REE patterns of sediment trap planktonic foraminifera exhibit a flat shape and lack a Ce anomaly (Ce/Ce*), indicating that pure foraminiferal calcite was analyzed (Fig. 3E). However, the REE patterns of sedimentary foraminiferal coatings from sediment core samples exhibit enrichment of light-REE (LREE) and middle-REE (MREE) in comparison to the heavy-REE (HREE) (Fig. 3D). Since the coating composition is much higher than the foraminiferal calcite, we henceforth refer to it only as “foraminiferal coatings”. Positive Ce/Ce* in benthic foraminiferal coatings, with values ranging from 1.23 and 1.57 (Fig. 4B), is found in all sedimentary foraminiferal coating patterns and is consistent with Ce scavenging from surface waters. During the Holocene of core CF10-01, middle-REE anomaly (MREE/MREE*) of benthic foraminiferal coatings fluctuate between 1.26 and 1.53, and the HREE/LREE varied between 0.41 and 0.68 (Fig. 4C,D).

The paleoproductivity proxy Benthic Foraminiferal Accumulation Rate (BFAR) shows millennial-scale variability ranging between 3400 to 741,000 ind cm^−2^ kyr^−1^, including high values centered at 8.4, 5.6, 5.0, 4.1, 2.6, 2.1, and 1.2 ka (Fig. 4A). A periodicity of 1.2 ka is significant at 95% confidence in the spectral analysis of the BFAR record (Supplementary Fig. S1). The Ce/Ce* values of benthic foraminiferal coatings are positively correlated to the variability of the paleoproductivity proxy (BFAR versus Ce/Ce*: *r*^2^ = 0.87, *p* < 0.001; Supplementary Fig. S2B). Peaks observed in the BFAR and Ce/Ce* records are also observed in the HREE/LREE record at virtually the same timing (Fig. 4). Despite the similarity observed between records of REE patterns and the BFAR records, similar peaks are not observed in the Mn/Ca record of benthic foraminifera coatings, which exhibits higher values only at 8.3 and 4.1 ka (0.12 and 0.10, respectively) (Fig. 4E).

### Stable isotopes and ε_Nd_ of foraminiferal authigenic oxide coatings

Stable carbon and oxygen isotopes (δ^13^C and δ^18^O) of epibenthic foraminifera *Cibicides kullenbergi* were relatively constant over the Holocene. The δ^13^C values vary between 0.57 and 1.48‰, with average value of 1.07 ± 0.15‰. The δ^18^O results vary between − 0.52 and 1.63‰, with an average value of 0.62 ± 0.22‰ (Fig. 4H, I). The ε_Nd_ signature of benthic foraminiferal coatings vary between –16.22 ± 0.33 and –15.73 ± 0.33, which is within the error (*t* = 0.86; *p* > 0.05) of planktonic foraminifera coatings ε_Nd_, which exhibited values between –16.69 ± 0.52 and –15.65 ± 0.29 (Fig. 4G).

## Discussion

Correlations between BFAR and particle reactive REE allow us to constrain past changes in the biological pump, how biological productivity, sediment flux and burial controlled marine carbon sequestration on millennial-scales, and how this in turn was related to global paleoclimate changes. We examine the delivery and preservation of organic carbon, and their associated REE, through the water column to shallow oxic depths in the sediment. We also constrain the chemical and biological processes which occur during vertical organic particle transport from the sea surface to the seafloor. Finally, we will link our biological and chemical records to other Holocene palaeoclimate records in order to examine interhemispheric patterns of millennial-scale variability in the Atlantic.

### REE in foraminiferal authigenic oxide coatings

The incorporation of trace elements in foraminifera is linked to the oxygen content of seawater in which calcification took place. Under oxic conditions, most of the elements incorporated into foraminifera calcite occur in low concentrations^34^ but higher elemental concentrations are instead present in microscale authigenic oxides coatings precipitated on the surfaces of foraminiferal shells^35,36^. Comparing sediment trap (i.e. F-150#3) foraminifera and sedimentary (i.e. CF10-01) foraminifera with authigenic oxide coatings we find that REE concentrations increase by up to tenfold in sedimentary planktonic and 14-fold in sedimentary benthic foraminifera relative to sediment trap planktonic foraminifera (Fig. 2). A similar increment of up to tenfold in REE concentration was obtained in planktonic foraminiferal coatings from a sediment core collected at 4.5 km water depth in the North Atlantic^35^, although the foraminifera showed higher absolute concentrations than those obtained in this work. The increase in trace elements and REE concentrations between sediment trap F-150#3 and sediment core CF10-01 samples provides evidence for the precipitation of authigenic oxides in foraminifera from core CF10-01 as coatings at the sediment–water interface.

The precipitation of foraminiferal authigenic oxide coatings occurs as a *post-mortem* process that involves the incorporation of trace elements (i.e. metals and REE) from bottom and pore waters into metal oxides, which precipitate under oxic conditions. The tight coupling of Mn and REE concentrations (*r*^2^ = 0.78, *p* < 0.001; Supplementary Fig. S2A) suggests that the REE are largely associated with authigenic Mn-oxides^35,36^.

Is the composition of REE in foraminiferal coatings controlled by redox changes in the sediment column? Considering the Ce/Ce* as a paleo-redox sensitive proxy^40^, our observation of high and positive Ce/Ce* values suggests that oxides precipitated and incorporated REE under fully oxic conditions. Importantly, the highest Ce/Ce* values match peaks in organic carbon export to the seafloor represented by the BFAR (Fig. 4 A,B). If the authigenic oxides had been formed under suboxic conditions or associated with more reducing conditions caused by increased organic carbon export, the Mn and Ce from oxides would have been reduced and mobilized into pore waters resulting in a decrease of Ce/Ce* in authigenic oxide coatings overprinting the seawater REE signal^37^. Instead, the authigenic oxides forming near the sediment–water interface record a seawater signature. This is supported by a study of sulphur cycling in the CFUS which also concluded that the low degree of pyritization, concentrations of pore water sulfide, and sedimentary reducible sulphur were caused by oxidation processes in the first centimeters of the sedimentary column^38^. Downcore measurements on CF10-01 consistently show low U/Ca values (Fig. 4F), which is a strong evidence against redox diagenesis controlling the REE patterns during the Holocene. Thus, we argue that the increased organic carbon export events were not strong enough to reduce the well-oxygenated conditions in the pore waters in this shallow site, but enhanced the positive Ce anomaly.

### Water column sources and cycling of REE

The source of REE to our study site potentially includes^35,37,39,40^: (i) reductive dissolution of authigenic oxides; (ii) waters mass advection; (iii) terrigenous inputs; and/or (iv) vertical cycling by particle reactive scavenging. Reductive dissolution of authigenic oxides in the water column can be ruled out because there is no strong oxygen minimum zone in the study area^41^. In addition, sulphur cycling in CFUS suggests oxic conditions of bottom waters and Holocene sediments^38^, as well as the strong positive Ce anomaly and the consistently low U/Ca values also do.

Changes in circulation and upwelling during the Holocene could have changed the REE pattern due to differences in water mass source at the study site, once surface water tend to exhibit lower HREE/LREE than subsurface water masses^22,42^. If HREE/LREE values were responding to water mass changes, then the higher HREE/LREE values (~ 3.94) could indicate a greater proportion of SACW because it would match the REE pattern of subsurface waters (around 400 m depth) from Brazil Basin^42^ (Fig. 3A). However, modern hydrographic observations^14^ and stable isotope records^16^ suggest no significant variability in bottom water mass proportion at the continental shelf and uppermost slope of the study location even when oceanographic changes occurred in the shallow depths (upwelling process). The modern signature of SACW (dissolved inorganic carbon δ^13^C = 1.30 ± 0.22‰; δ^18^O = 0.46 ± 0.10‰)^43^ is similar to the values reconstructed for the last two centuries in the area. While δ^13^C and δ^18^O mean values for the recent and sub-recent epibenthic foraminifera *Cibicides kullenbergi* are around 1.04 ± 0.10‰ and 0.65 ± 0.24‰^16^ respectively, our Holocene epibenthic mean δ^13^C (1.07 ± 0.15‰) and δ^18^O (0.62 ± 0.22‰) are virtually the same. Although we cannot discard additional influences over the *C. kullenbergi* δ^13^C signature (e.g., phytodetritus), the constant long-term trends of epibenthic foraminiferal δ^13^C and δ^18^O suggest that modern bottom water circulation regime should have persisted throughout the Holocene (Fig. 4H,I).

Considering the shallow depth and near-shore location of core CF10-01, it is possible that the source of REE could be riverine input. Changes in terrestrial inputs could have changed the MREE/MREE*, which its increment under oxic conditions is often related to input from terrestrial REE sources^37^. Indeed, the MREE/MREE* values above 1 may indicate the Paraíba do Sul River influence at our study site, but the variability is relatively constant over the Holocene. The *ε*_Nd_ values analyzed in both planktonic and benthic foraminiferal coatings show non-radiogenic values varying between –16.69 and –15.64 (Fig. 4G). These *ε*_Nd_ values resemble the signature of the recent and Holocene sediments nearby core CF10-01, which show non-radiogenic values from −17.5^30^ to −14.0^31^. Our *ε*_Nd_ results from core CF10-01 are consistent with SACW circulation dominating the core site and indicate inputs of unradiogenic continental detritus, as also suggested by a GEOTRACES study of seawater along the South American continental margin^22^. The unradiogenic *ε*_Nd_ values of foraminiferal authigenic oxide coatings from core CF10-01 suggest a continental detritus signature overprinting the seawater *ε*_Nd_ signature. However, although the *ε*_Nd_ and the MREE/MREE* values suggest a terrestrial influence on the seawater composition recorded by foraminiferal authigenic oxide coatings, the MREE/MREE* variability is not similar to the Ce/Ce* and HREE/LREE records throughout the Holocene (Fig. 4B–D). The MREE/MREE* values have no correlation to the HREE/LREE (*r*^*2*^ = −0.13*; p* > 0.05) and present a negligible correlation to the Ce/Ce* (*r*^*2*^ = −0.24*; p* = 0.05). This suggests that the Ce/Ce* and HREE/LREE variabilities were likely caused by a different process other than changes in terrestrial input.

The transport of REE through the water column likely involves reversible scavenging from the water column by the particulate organic carbon and its release to sediment pore waters by post-depositional organic carbon oxidation. To analyze this possibility, we compared the REE patterns to the paleoproductivity proxy BFAR. We observed that BFAR and REE patterns are strongly and positively correlated (*r*^*2*^ = 0.83*; p* < 0.01 for BFAR vs. Ce/Ce*, and *r*^*2*^ = 0.50*; p* < 0.05 for BFAR vs. HREE/LREE) (Supplementary Fig. S2B,C). Considering the coupled variability between our organic carbon export proxy and REE patterns, we propose that the REE composition of foraminiferal authigenic oxide coatings was dominantly controlled by particle-reactive cycling. The potential of different scavenging phases to produce the REE patterns in the ocean has been examined by some recent models^18,21,37^, but these studies were unable to reproduce the main phase driving REE patterns in shallow waters. Our data show that the normalized REE pattern of foraminiferal coatings from core CF10-01 is more similar in shape as a typical sedimentary organic carbon REE pattern in the Atlantic Ocean than a typical Fe–Mn oxides pattern^19^ (Fig. 3B–D) suggesting that particulate organic carbon are the most likely carrier phase at the depths of our study sites (< 200 m water depth).

### Coupled changes in marine carbon sequestration and REE cycling

The paleoproductivity proxy BFAR is widely used to reconstruct past organic carbon export to the seafloor^6,16,44,45^ by the increments of primary production^46^. This assumption is also reasonable for the CFUS due to the predominance of autochthonous marine organic carbon in the sediment as a result of the upwelling^28,29^. In these shallow depths, the carbon sequestered by primary producers is transferred to the seafloor and increases the density of benthic foraminifera by increasing food availability to benthic organisms. The export of fresh organic carbon particularly benefits benthic opportunistic species which reproduce quickly under the seasonal high-primary productivity events typical of the CFUS (e.g., *Globocassidulina subglobosa*)^16^. Even though the high accumulation of benthic foraminifera does not indicate the length of organic carbon in the sediments, the BFAR is a strong record of the marine carbon sequestration in millennial-scale during the Holocene.

During periods of high BFAR, the REE patterns of foraminiferal authigenic oxide coatings have a higher Ce/Ce* and HREE/LREE values in comparison to the periods of low organic carbon export (i.e. low BFAR) (Fig. 4). The association of a scavenged REE pattern during higher productivity events indicates that organic particles sinking in the water column are responsible for scavenging REE from shallower depths to the seafloor. Thus, the positive peaks in the Ce/Ce* record are the result of enhanced Ce removal from seawater during the vertical transport of organic carbon to the seafloor^19,39^. The scavenged Ce might have been released into pore waters during partial oxidation of organic carbon^20^ under oxic conditions, so that they are directly incorporated into authigenic oxide coatings. This is an evidence of the potential of the marine organic carbon to scavenge dissolved REE in surface and shallow waters and export them to the greater depths, where they can be released to the seawater impacting its signature.

The REE patterns in foraminiferal authigenic oxide coatings from shallow sediment cores are not integrating the REE scavenged through the water column^37^ but instead are reflecting the bottom water mass REE pattern, which is set by vertical export of organic carbon. This also means that, in shallow depths, changes in the Ce/Ce* and HREE/LREE can be used as proxies for marine carbon sequestration by the biological pump. A similar suggestion was made in a GEOTRACES section across the subtropical South Atlantic (at 40^o^S), where the scavenging dominates desorption at shallow depths (i.e. above 200 m) and the influence of horizontal water mass mixing is only observed in depths below 200 m^22^. Scavenging removes the LREE from the solution and transfer it to the organic carbon thereby changing the shallow water pattern to high HREE/LREE. Our observation of raised HREE/LREE scavenged pattern and the addition of LREE (especially Ce) recorded by foraminiferal authigenic oxide coatings during periods of high BFAR values suggests its use as a proxy for organic carbon oxidation in the sediment. It also suggests how biological processes can affect Nd cycling in the upper ocean both spatially and though time. The Nd isotope signature of surface and sub-thermocline water masses might be modified by the biological productivity and perhaps suggests a reason why Nd isotopes mimic nutrient tracers in some opportunities^47^.

### Ocean–atmosphere control over marine carbon sequestration

**Figure 5:**
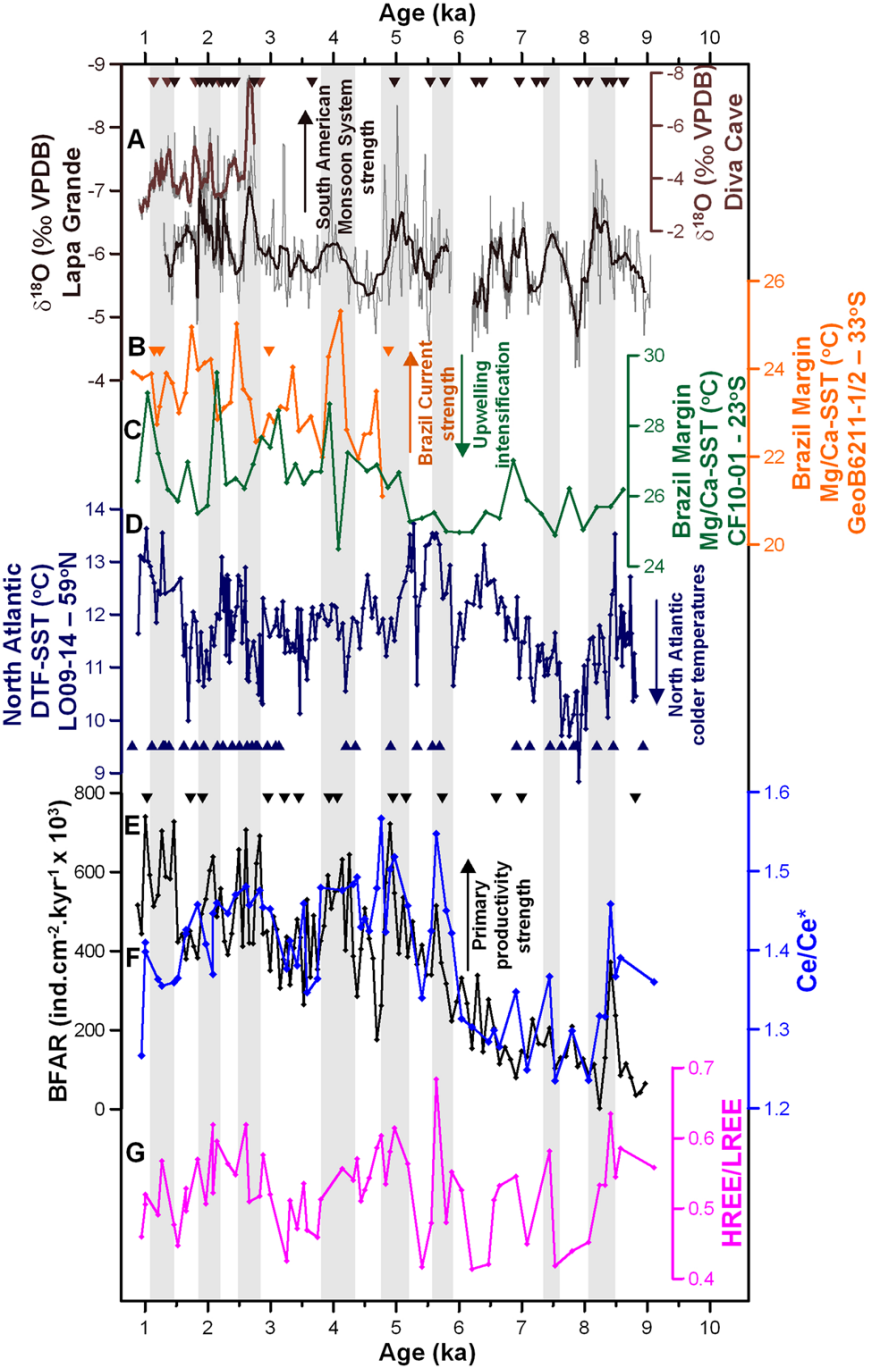
Records of organic carbon export and REE patterns compared to ocean and climate records. **(A)** δ^18^O (‰) from speleothems records under influence of the SAMS (Lapa Grande^10^: black; Diva cave^11^: brown), **(B)** Mg/Ca SST of *Globigerinoides ruber* (white) from the sediment core GeoB6211-1/2, Brazil Margin^8^ (orange), **(C)** Mg/Ca SST of *Globigerinoides ruber* (white) from the sediment core CF10-01, Brazil Margin^17^ (green), **(D)** diatom transfer function SST from the sediment core LO09-14, North Atlantic^5^ (dark blue), **(E)** BFAR record from core CF10-01, Brazil Margin (black; this study), (F) Ce anomaly of benthic foraminiferal coatings from core CF10-01, Brazil Margin (light blue; this study), and **(G)** HREE/LREE of benthic foraminiferal coatings from core CF10-01 from Brazil Margin (purple; this study). Triangles referent to U/Th dates for speleothems from Lapa Grande (dark brown) and Diva Cave (light brown) and to radiocarbon dates for GeoB6211-1/2^8^ (orange), LO09-14^5^ (dark blue), and CF10-01^17^ (black). Gray bars highlight periods of SAMS intensification based on BFAR and increased particle reactive element delivery to the seafloor.

The long-term trends registered in BFAR and Ce/Ce* matches the SST trend in core CF10-01 (Fig. 5C,E,F), suggesting a common oceanographic forcing operating during the early and mid-Holocene. We propose that the offshore location of the Brazil Current during the early Holocene reduced the export of labile organic carbon to the seafloor (reduced BC-driven upwelling) until approximately 5.5 ka at our site, as a consequence of lower than modern sea-level during the early Holocene^48^. Considering a scenario with lower productivity, we would expect lower Ce/Ce* and HREE/LREE values during the early and mid-Holocene. However, HREE/LREE do not change to the same extent as the Ce/Ce* values during productivity events and very low values are not observed during these periods of lower productivity. These results mean that particulate organic carbon is the preferential phase controlling excess Ce removal from seawater but is not the only phase responsible for the fractionation of the HREE/LREE. The HREE/LREE was fractionated by all sediments (e.g., Fe/Mn oxyhydroxides, calcium carbonate, and other inorganic sediment particles^37,39^) which are always abundant in the location independent of the amount of particulate organic carbon. Therefore, changes in Ce/Ce* are caused by millennial-scale variability of organic carbon which contribute towards but are in addition to background fractionation of HREE/LREE by all particle types.

The short-term fluctuations in BFAR are coupled to both HREE/LREE and Ce/Ce* records, suggesting the occurrence of enhanced organic carbon export to the seafloor during periods of rapid Holocene climate change. Rapid increases in the organic carbon export are also coincident with periods of a strengthened BC, as our BFAR, HREE/LREE, and Ce/Ce* peaks closely match positive SST anomalies in the BC^8^ (Fig. 5B,E,F,G). SST peaks from core GeoB6211-1/2 (655 m water depth at 32^o^S) are antiphase to SST from CF10-01^17^ (Fig. 5C), confirming that BC strength and its offshore displacement results in an intensification of the BC-driven upwelling, which brings cold and nutrient-rich waters to the surface, stimulating biological productivity, the biological pump, and carbon sequestration.

Our proxy records of changes along the Brazilian margin correspond to precipitation anomalies recorded in speleothem δ^18^O records from central-eastern Brazil^10,11^ related to the SAMS strength (Fig. 5A). We interpret this correspondence as indicating the NE wind pattern controlling changes in CFUS upwelling, marine biological productivity, marine organic carbon export, as well as continental rainfall. While SAMS and NE winds affect continental rainfall and river outflow, this has a less direct influence on integrated river outflow and the flux of suspended sediment as well as the arrival of river and sediment plumes at our outer shelf core site. We compared our MREE/MREE* to another fluvial proxy reconstructed from a nearby core, the clay mineral data from core CF02-02 located at 124 m water depth, and illite abundance records^49^ showed a similar trend as the MREE/MREE* from core CF10-01 (Supplementary Fig. S3D,E). However, both MREE/MREE* and illite diverge from BFAR, Ce/Ce*, and HREE/LREE records (Supplementary Fig. S3) and also do not show statistical correlation (BFAR versus MREE/MREE*, *r*^*2*^ = −0.29; *p* > 0.05; Supplementary Fig. S2D). The shift towards less fluvial influence in both the clay mineralogy and MREE/MREE* records around 5.5 ka, as well as the absence of MREE/MREE* fractionation in millennial-scale suggest that the Paraíba do Sul River plume was trapped into coastal areas^30^ during the late Holocene. The Brazil Current inshore position was the mechanism responsible for it, which did not allow a substantial portion of these sediments to reach the outer shelf. However, the continental signature (predominantly from Paraíba do Sul River) was still transported to our site by longshore drift^50^, which is then evidenced by our MREE/MREE* and unradiogenic ε_Nd_ values without the SAMS variability imprint.

Millennial-scale variability of organic carbon export as recorded in our BFAR, HREE/LREE, and Ce/Ce* records is not only similar to SAMS oscillation but also to surface ocean circulation and climate reconstructions from the Northern Hemisphere^4,5,9,10^. Considering that the temporal resolution of the BFAR record is not appropriate to track decadal-scale changes but is adequate for millennial-scale changes, we suggest that the millennial-scale climatic variations observed along the Brazilian margin and eastern South America (Fig. 5A,B,E,F) are related to a common interhemispheric ocean–atmosphere mechanism that persisted throughout the Holocene.

The most likely candidate for increasing SAMS strength on this timescale is the southward shift of the ITCZ as a result of asymmetric temperature change between the North and the South Hemisphere. Indeed, the SAMS was enhanced during Holocene periods of reduced cross-equatorial heat transport, resulting in the North Atlantic SST cooling and South Atlantic SST warming^10,11^. ITCZ southward displacements were related to the intensification of the NE trade winds, that caused the intensification of the SAMS^10^. The same intensification of the NE trade winds prevented the cross-equatorial northwards heat transport by weakening the North Brazil Current^9^. This reduction of the cross-equatorial heat transport increased western South Atlantic SST and strengthened the BC^8,9,51^, enhancing the BC-driven upwelling and organic carbon export to the seafloor that resulted in the BFAR high values registered by core CF10-01. High BFAR and low speleothem δ^18^O values correspond to North Atlantic SST shifts to colder temperatures as reconstructed from core LO09-14 collected at 59^o^N^5^ (1,685 water depth), especially around the ages 4.9, 4.2, 2.7, and 2.0 ka (Fig. 5D).

Although it is unclear whether there were changes in Atlantic Meridional Overturning Circulation during the mid-late Holocene^52–54^, our South Atlantic records show a strong connection to North Atlantic climate variability. The good match between terrestrial and marine records suggests that the CFUS records are part of a regional climate mode in the western South Atlantic and eastern South America that is very sensitive to the millennial-scale North Atlantic cold conditions. The variability shown at the Brazil margin and the continental records suggest a direct link to the North Atlantic via atmospheric and oceanic changes associated with the ITCZ. Moreover, the coupled changes in BFAR and REE chemistry show that these climate changes forced marine carbon sequestration in the tropics. The large spatial extent of shallow tropical marine environments and the hotspots of biological productivity occurring there, enhance their ability to affect the global carbon cycle. This study shows the continental shelf ability to sequester carbon on rapid timescales and highlights the importance of understanding atmospheric teleconnection between the polar regions and the tropics.

## Conclusion

High temporal resolution reconstruction of biological productivity proxy BFAR and REE records of foraminiferal authigenic oxide coatings from the shallow depth sediment core CF10-01 from the western South Atlantic are interpreted as millennial-scale records of changes in marine carbon sequestration throughout the Holocene. The comparison between foraminifera from core CF10-01 and sediment trap foraminifera supports *post-mortem* precipitation of authigenic oxides as foraminiferal coatings. Although MREE/MREE* and *ε*_Nd_ data point to a terrestrial input of REE to the study site, the variability of the HREE/LREE and Ce/Ce* values record of past changes in organic carbon production and export to the seafloor in the continental shelf of the western South Atlantic. Our Ce/Ce*, HREE/LREE, and BFAR records are coherent with increased particulate organic carbon scavenging of REE followed by its export to the seafloor during times of high primary productivity. Taken together, BFAR and REE patterns can be used as a proxy to reconstruct carbon sequestration. The comparison to North Atlantic SST records shows a strong link to SAMS strength and suggests a common interhemispheric ocean–atmosphere system liable of changing on millennial-scale which involve physical oceanographic, marine biological, and chemical cycling in continental shelf regions. Shallow marine depths appear to be a dynamic part of the marine carbon sequestration system and are tightly coupled with regional and global rapid climate changes.

## Material and methods

### Sediment trap and sediment core foraminifera sampling

Samples from a moored F-150#3 sediment trap deployed at the shelf edge were collected during the austral winter in 2011 at 23°36′ S, 41°34′ W^55^ (Fig. 1). All mixed planktonic foraminifera species larger than 150 μm were picked and merged into two samples corresponding to the sediment trap depths (50 and 100 m). Samples from trap cups were stored in a buffered formaldehyde solution (4%) with adjusted salinity to 70 PSU.

Foraminifera samples from the sediment core CF10-01 at 23°24′ S, 41°39′ W (128 m water depth) were analyzed for the last ca. 9 ka (Fig. 1). Bulk sediment samples were wet sieved at 63 μm and the remained foraminifera larger than 150 μm were picked for chemistry analyses. For the benthic foraminifera paleoproductivity proxy, foraminifera larger than 63 μm were picked in a 2 cm-resolution.

### Age model

The analyzed section of the sediment core CF10-01 has the chronology obtained by the 16 radiocarbon ages on organic carbon measured by accelerator mass spectrometry (AMS)^17^. The choice of organic carbon for dating occurred because of the lack of enough planktonic foraminifera tests and the predominance of autochthonous marine organics due to the upwelling process^28,29^. The age model was previously published^17^ and has been improved using the calibration curve Marine 09 and a reservoir effect of 8 ± 17 years^56^.

### Benthic foraminifera paleoproductivity proxy

To reconstruct past changes in benthic conditions and fauna, the Benthic Foraminifera Accumulation Rate (BFAR) was applied. The sedimentation rate previously determined^17^ was applied here to calculate the BFAR as a product of the sedimentation rate, dry bulk density, and benthic foraminifera density^46^. REDFIT spectral analysis of the linear detrended BFAR values was performed with the PAST software^57^ for the last 6 ka, once it is a time-period not affected by the glacial boundary conditions^5^.

### Stable isotopes measurements

Stable carbon and oxygen isotopes (δ^13^C and δ^18^O) were measured in 10 to 15 specimens of benthic foraminifera *Cibicides kullenbergi* Parker, 1953 larger than 150 μm. Analyses were performed in a Finning MAT 252 spectrometer at the University of Davis, considering a precision of ± 0.03‰ and ± 0.07‰ for δ^13^C and δ^18^O, respectively. Samples were reacted in supersaturated 105% H_3_PO_4_ at 90 °C using a Gilson Multicarbon Autosampler. The data were adjusted to Vienna Pee Dee Belemnite (VPDB) using NBS-19 calcite standard. Bracketing stable isotope results correspond to average ± standard deviation.

### Foraminifera element concentrations and neodymium isotopes

Two sediment trap mixed planktonic foraminifera samples were oxidatively cleaned prior to the analyses to remove organics^58^. Sediment core samples composed of mixed hyaline benthic and mixed planktonic foraminifera from the sediment core CF10-01 were crushed between glass plates and clays were removed by ultrasonic bathing without removal of the authigenic oxide coatings. The samples were dissolved in 0.1 M acetic acid and transferred to Teflon vials after being centrifuged.

Foraminiferal element concentrations were analyzed on 5% aliquots after being dried, re-dissolved in 0.3 M HNO_3_. Trace element concentrations measured in a Perkin-Elmer SCIEX Elan DRC II quadrupole inductively-coupled mass spectrometer. The Ca concentration of each sample was prior analyzed before diluting and running all samples and standards at 100 ppm Ca^59^. The ratio between heavy and light REE (HREE/LREE) was calculated as the ratio between Yb and Nd concentrations (ppm/ppm). Cerium (Ce/Ce*) anomaly considered normalized PAAS values^60^ and was calculated as 3*Ce/(2*La + Nd)^61^. The middle-REE enrichment (MREE/MREE*) was calculated as [(Gd + Dy)/2]/[(Nd + Yb)/2]^62^ normalized to PAAS values.

For the neodymium isotopes (ε_Nd_) in benthic and planktonic foraminifera samples, the REE were separated from other elements using Eichrom TRUspec™ resin, and neodymium was extracted from other REE using Eichrom LNspec™ resin. Samples were analyzed in Neptune Plus multi-collector mass spectrometer (MC-ICP-MS) at the University of Cambridge. Measurements were corrected for mass fractioning to a ^146^Nd/^144^Nd ratio of 0.7219. Samples were bracketed with concentration matched solutions of standard JNdi-1 with value of 0.512115 ± 7^63^. Error bars correspond to 2*σ* of combined internal and external reproducibility of the bracketing standards.

## Supplementary Information

Supplementary Figures.

## Data Availability

Data from this work is available through PANGAEA database at https://doi.pangaea.de/10.1594/PANGAEA.931861.
