# Supplementary material for: Coupled changes in western South Atlantic carbon sequestration and particle reactive element cycling during millennial-scale Holocene climate variability
Source: Sci Rep. 2021 Dec 21;11:24378. doi: 10.1038/s41598-021-03821-8 (PMC8692323; doi:10.1038/s41598-021-03821-8)
Supplement: Supplementary file 1 — Supplementary Figures. [file 41598_2021_3821_MOESM1_ESM.pdf]

Coupled changes in western South Atlantic carbon sequestration and particle reactive element cycling during millennial-scale Holocene climate variability

Bruna B. Dias [1,2*], Alexander M. Piotrowski [3], Cátia F. Barbosa [1], Igor M. Venancio [1,4], Cristiano M. Chiessi [2], Ana Luiza S. Albuquerque [1]

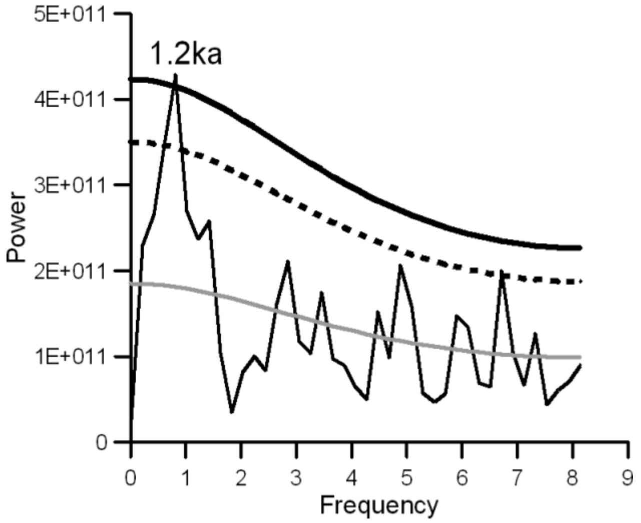

Figure S1. Spectral analysis of Benthic Foraminifera Accumulation Rate (BFAR) for the last 6.0 ka from core CF10-01. Black and dashed lines indicate 95% and 90% significance levels, respectively. Grey line is AR(1).

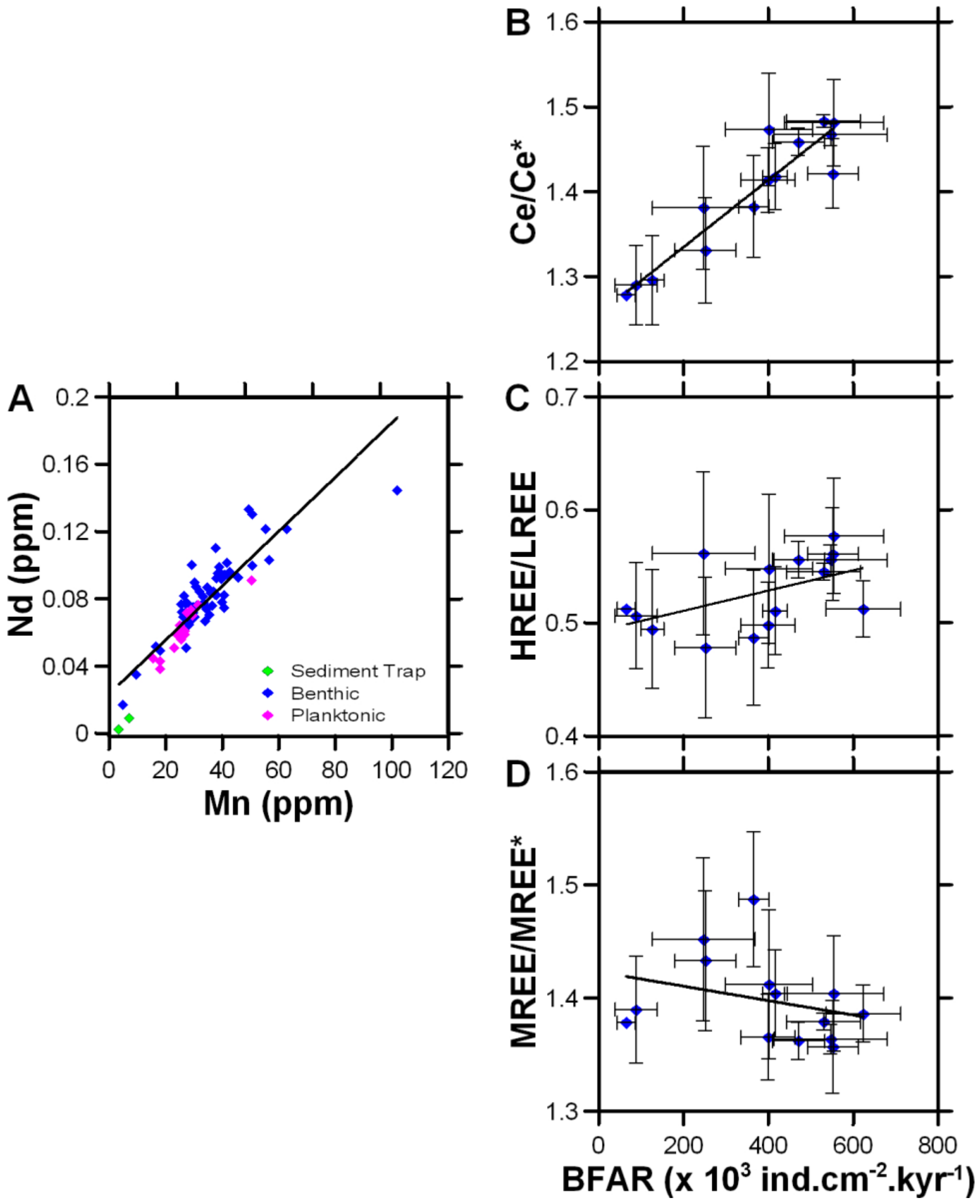

Figure S2. Relations between: (A) Mn and Nd concentrations of sediment trap planktonic foraminifera (green) and foraminiferal authigenic oxide coatings, considering benthic foraminiferal coatings values (blue) and planktonic foraminiferal coatings values (purple); (B) reconstructed Ce anomaly (Ce/Ce*) of benthic foraminiferal coatings and BFAR index; (C) reconstructed HREE/LREE values of benthic foraminiferal coatings and BFAR index; (D) reconstructed MREE/MREE* values of benthic foraminifera coatings and BFAR index.

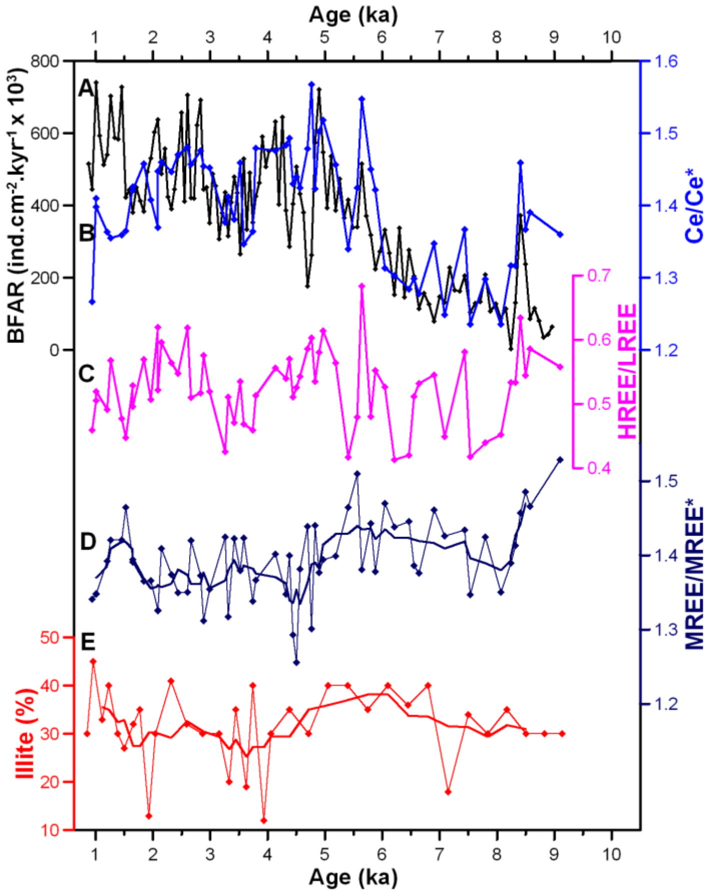

Figure S3. Records of organic carbon export and REE patterns compared to illite abundance in CFUS. (A) BFAR record from core CF10-01, Brazil Margin (black; this study), (B) Ce anomaly (Ce/Ce*) of benthic foraminiferal coatings from core CF10-01, Brazil Margin (light blue; this study), (C) HREE/LREE of benthic foraminiferal coatings from core CF10-01, Brazil Margin (purple; this study), (D) Middle-REE enrichment (MREE/MREE*) of benthic foraminiferal coatings from core CF10-01, Brazil Margin (dark blue; this study), and (E) illite abundance from the sediment core CF02-02, Brazil Margin (red; Albuquerque *et al.*, 2016).

Reference

Albuquerque, A.L.S., *et al.* Mineral and elemental indicators of post-glacial changes in sediment delivery and deposition under a western boundary upwelling system (Cabo Frio, southeastern Brazil). *Palaeogeogr. Palaeoclimatol. Palaeoecol.* **445**, 72–82. https://doi.org/10.1016/j.palaeo.2016.01.006 (2016).
